# Traditional Chinese Medicine Formulas in Delaying Aging: From Theoretical Foundations to Molecular Mechanisms and Translational Perspectives

**DOI:** 10.1111/jcmm.71371

**Published:** 2026-09-28

**Authors:** Chenrong Jin, Ding Du, Xiaorui Yu, Xiaoyan Ji, Xiuyun Zhang, Anning Li, Shuang Zu, Bo Qu, Daqing Zhao, Dianpeng Wu, Meichen Liu

**Affiliations:** ^1^ Northeast Asian Institute of Traditional Chinese Medicine, Jilin Provincial Key Laboratory for Efficacy Research and Utilization of Characteristic Traditional Chinese Medicine Changchun University of Chinese Medicine Changchun China; ^2^ Chengdu Wenjiang Yujunfang Internet Hospital Co., Ltd. Chengdu China; ^3^ Jilin Aodong Yanbian Pharmaceutical Co., Ltd. Dunhua China

**Keywords:** aging biomarkers, anti‐aging, cellular senescence, mitophagy, multi‐omics integration, network pharmacology, traditional Chinese medicine formula

## Abstract

Aging is a systemic decline in physiological integrity, driving chronic diseases such as neurodegeneration, cardiovascular disorders, and metabolic syndromes. Rapid global population aging urgently demands effective interventions. Traditional Chinese medicine (TCM) formulas, characterized by multi‐component, multi‐target, and multi‐pathway synergy, offer a promising paradigm for delaying aging. This review systematically integrates recent advances in TCM formula‐based anti‐aging research, bridging classical TCM gerontology with the 14 modern hallmarks of aging. We categorize representative formulas into four mechanistic groups targeting mitochondrial homeostasis and autophagy, cellular senescence and inflammatory microenvironment, stem cell maintenance and epigenetic reprogramming, and gut microbiota and systemic metabolism, and explore their holistic efficacy, key bioactive constituents, and multi‐component synergy. In terms of methodological innovations, we introduced network pharmacology combined with AI‐driven target screening and proteomic aging clocks to assess biological age. We discussed challenges in chemical complexity, mechanistic completeness, and clinical translation, and proposed a roadmap for development. This review establishes an integrative model that integrates TCM principles with modern aging biology, offering insights for translational research toward healthy longevity.

**Abbreviations**
AD: Alzheimer's disease
BMSCs: Bone marrow mesenchymal stem cells
BYHWD: Buyang Huanwu Decoction
BZBC: Bazi Bushen capsules

*C. elegans*

:
*Caenorhabditis elegans*

GEG: Guilu Erxian Glue
GQS: Guiqi Shen
HSCs: Haematopoietic stem cells
KXS: Kaixin San
LWDHW: Liuwei Dihuang Wan
SASP: Senescence‐associated secretory phenotype
SA‐β‐gal: Senescence‐associated β‐galactosidase
SJZD: Sijunzi Decoction
TCM: Traditional Chinese medicine
TMA: Trimethylamine
YGP: Yougui Pill
YSHSG: Yishen Huashi Granule
ZGP: Zuogui Pill

## 1 Introduction

Population aging is becoming the most severe global challenge of the 21st century [1]. The World Health Organization predicts that by 2030, the global population aged 60 and above will exceed 1.4 billion, and by 2050, this number will reach 2.1 billion [2, 3]. Aging is not a single event, but a complex biological process driven by a series of interrelated molecular dysbiosis, protein homeostasis loss, chronic inflammation, oxidative stress, mitochondrial dysfunction, cellular aging, and nutrient perception disorders, which collectively accelerate the decline of body function and significantly increase susceptibility to age‐related diseases such as neurological, cardiovascular, metabolic, musculoskeletal, and immune system disorders [4, 5]. In recent years, some synthetic drugs have shown anti‐aging potential in preclinical and clinical studies, such as metformin, which can prolong the lifespan of model organisms and improve human health [6, 7]. However, these drugs generally face bottlenecks such as unclear long‐term safety and low preventive acceptance among healthy populations, and their single target mode of action is difficult to cope with the systemic characteristics of the aging network [7, 8].

TCM formulas supported by thousands of years of evidence‐based medical practice and centred on holistic regulation and individualized treatment, simultaneously modulate multiple hallmarks of aging through synergistic interactions among their diverse bioactive constituents—a paradigm increasingly aligned with modern geroscience [7, 9, 10]. A recent study in ‘*Cell Metabolism’* used machine learning to identify blood‐based aging protein features that robustly predict frailty and inflammatory states [11]. This progress covers the characteristics of multi‐system aging networks and is highly consistent with the mode of action of TCM, which involves multiple components, targets, and overall regulation. It provides a feasible scientific framework for the systematic evaluation of the anti‐aging effects of TCM prescriptions. Accumulating evidence indicates that representative TCM formulas, such as Buyang Huanwu Decoction (BYHWD), Bazi Bushen Capsules (BZBC), and Guiqi Shen (GQS) Oral Liquid, exert systemic anti‐aging effects across multiple organ systems and hallmarks. They have been reported to modulate up to 12 aging hallmarks, ranging from genomic instability to cellular senescence, achieving holistic regulation beyond the reach of single‐target agents [12, 13, 14, 15, 16, 17, 18, 19, 20, 21].

Although research on TCM anti‐aging is becoming increasingly active, reviews that organically integrate classical TCM theories with modern molecular mechanisms remain scarce. To address this gap, this review maps TCM gerontology theories onto 14 well‐established aging hallmarks from a precision‐medicine perspective. Based on the core biological signatures regulated by each formula, we categorize classic TCM formulas into four groups that respectively target mitochondrial homeostasis and autophagy, cellular senescence and the inflammatory microenvironment, stem cell maintenance and epigenetic reprogramming, and gut microbiota and systemic metabolism, thereby bridging empirical TCM with mechanistic research. Methodologically, we employ AI‐driven network pharmacology, proteomic aging clocks, and multi‐omics integration to convert empirical evidence into testable outcomes. Translationally, we propose a clinical paradigm combining proteomic profiling, TCM constitution typing, and epigenetic age reversal as core endpoints, aiming to facilitate regulatory‐compliant anti‐aging trials and provide references for healthspan extension.

## 2 Methods

### 2.1 Database and Search Strategies

A comprehensive literature search was performed across five electronic databases: Web of Science, ScienceDirect, Google Scholar, PubMed, and NCBI, covering studies published from January 1995 to May 2026, with particular emphasis on cutting‐edge advances from 2024 to 2026. Search terms included combinations of ‘aging’, ‘anti‐aging’, ‘senescence’, ‘cellular senescence’, ‘traditional Chinese medicine formula’, ‘TCM decoction’, ‘herbal formula’, as well as specific formula names such as ‘Zuogui Pill’, ‘Yougui Pill’, ‘Bazi Bushen Capsules’, ‘Buyang Huanwu Decoction’ and ‘Liuwei Dihuang Wan.’ Two reviewers independently performed the search. After duplicates were removed, an initial screening of titles and abstracts was conducted to assess eligibility, followed by full‐text evaluation for potentially relevant articles. Disagreements during study selection were resolved through consensus discussion or, if necessary, consultation with a third reviewer.

### 2.2 Eligibility Criteria

Studies were included if they met the following criteria: (1) Original research articles published in English or Chinese; (2) Experimental investigations (in vivo, in vitro, or in silico) providing evidence of anti‐aging activity of TCM formulas or their bioactive constituents; (3) Assessment of anti‐aging effects using commonly accepted biomarkers, including but not limited to senescence‐associated β‐galactosidase (SA‐β‐gal) activity, senescence‐associated secretory phenotype (SASP), cell cycle regulators (p16, p21, p53), telomerase activity, oxidative stress markers (ROS, MDA, SOD), mitophagy‐and autophagy‐related proteins (PINK1, Parkin, LC3B, Beclin1), inflammatory cytokines (IL‐1β, TNF‐α, NLRP3), epigenetic or proteomic aging clocks, circulating senescence‐associated proteins (e.g., GAS6, GPNMB), and extracellular matrix remodelling indicators. Exclusion criteria were: (1) Reviews, conference abstracts, editorials, and unpublished data; (2) Studies unrelated to TCM formulas or lacking specific anti‐aging outcome measures.

### 2.3 Study Selection and Data Extraction

For all included studies, the following data were extracted: first author's name, year of publication, TCM formula or active compound, experimental model (in vitro, in vivo), dosage and treatment regimen, assessed aging hallmarks or biomarkers, key anti‐aging mechanisms, and reported outcomes regarding healthspan or lifespan extension.

## 3 Mapping TCM of Aging Onto Modern Precision Medicine Biomarkers

### 3.1 Classical TCM Theories of Aging

TCM views aging through a holistic, system‐based framework developed over two millennia. Its core view is that aging results from the gradual depletion of essential substances—*jing* (essence), *qi* (vital energy), *xue* (blood), and *jin‐ye* (body fluids)—alongside the natural decline of *zang‐fu* organ functions [22, 23, 24] (Figure 1). Among the various theories, the ‘Kidney Essence Depletion Theory’ is the most influential and widely accepted paradigm. This theory states that the kidney stores congenital essence, which forms the material basis for growth, development, reproduction, and ultimately the aging process itself [22, 25]. As *kidney essence* progressively declines with age, it leads to systemic functional deterioration, including reduced fertility, bone fragility, cognitive impairment, and loss of vitality [26, 27, 28].

**FIGURE 1:**
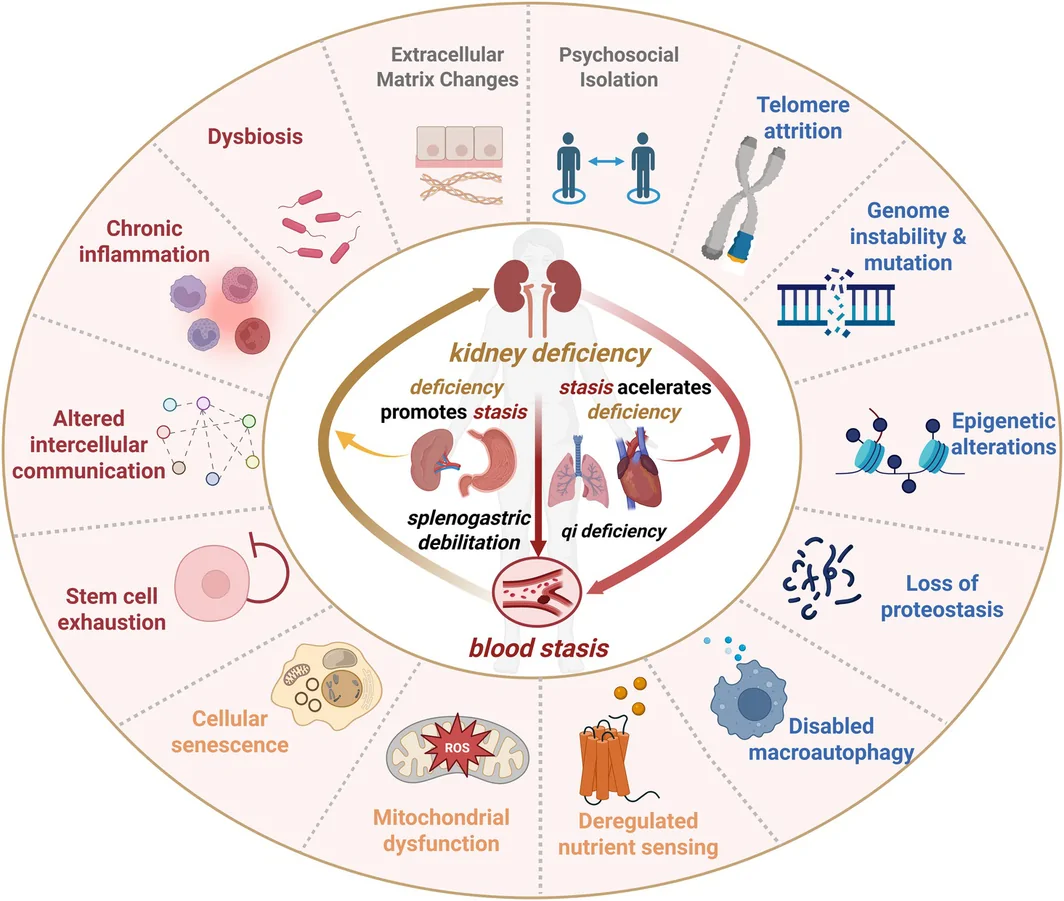
Aging theory from the perspectives of TCM and modern medicine. The schematic illustrates the TCM theories of aging and their related biological foundations. *Kidney deficiency* and *blood stasis* interact in a bidirectional vicious cycle, where *deficiency* promotes *stasis*, and *stasis* exacerbates *deficiency*. *Qi deficiency* and *spleen‐stomach debilitation* synergize with this process: Insufficient *qi* transforming power aggravates *blood stasis*, while splenogastric weakness impairs transportation and transformation, cuts off the source of *qi* and *blood* production, and accelerates multi‐organ decline. This core pathological axis is closely linked to a series of canonical hallmarks of aging, including telomere attrition, genome instability, epigenetic alterations, loss of proteostasis, disabled macroautophagy, dysregulated nutrient sensing, mitochondrial dysfunction, cellular senescence, stem cell exhaustion, altered intercellular communication, chronic inflammation, dysbiosis, extracellular matrix changes, and psychosocial isolation, which collectively mediate aging‐related pathophysiological processes.

‘Kidney Deficiency and Blood Stasis Theory’ concept has emerged as a more comprehensive model that integrates both *deficiency* and stagnation pathologies. In this view, depletion of *kidney essence* compromises the generation and circulation of *blood*, leading to microcirculatory stasis that further accelerates tissue degeneration. This bidirectional relationship—deficiency‐promoting *stasis*, and *stasis* exacerbating *deficiency—creates* a self‐perpetuating cycle that underlies the progressive nature of aging [29, 30].

Other perspectives, including the ‘*Qi‐Blood Theory*’ and the ‘*Spleen‐Stomach Theory*’, offer complementary insights. The ‘*Qi‐Blood Theory*’ posits that *qi* and *blood* constitute the driving force of life activities; their transition from prosperity to decline results in insufficient motive power, which aggravates *blood stasis* and forms a vicious cycle that ultimately triggers systemic functional deterioration. The ‘*Spleen‐Stomach Theory*’ focuses on the acquisition and transformation of acquired essence, holding that splenogastric debilitation impairs transportation and transformation, cuts off the source of *qi* and *blood* generation, deprives the viscera of nourishment, and thereby accelerates multi‐organ aging [31, 32]. The ‘Heart‐Kidney Interaction Theory’ emphasizes the role of dynamic balance between these two organs in sustaining youthful function, while the ‘*Liver‐Governing‐Dispersion Theory*’ highlights the significance of emotional regulation and unobstructed *qi* movement in delaying aging [33]. Collectively, these theories constitute the multidimensional cognitive framework of Chinese medicine gerontology.

### 3.2 Quantifying Aging Through a Precision Medicine Lens: The 14 Hallmarks of Aging and the Application of Proteomic Clocks

The conceptualisation of aging as a process driven by interconnected molecular features, first articulated and subsequently expanded by López‐Otín and colleagues, provides a tractable framework for aging research and therapeutic development [5, 34]. These hallmarks include genomic instability, telomere attrition, epigenetic alterations, loss of proteostasis, disabled macroautophagy, deregulated nutrient sensing, mitochondrial dysfunction, cellular senescence, stem cell exhaustion, altered intercellular communication, chronic inflammation, dysbiosis, changes in the extracellular matrix, and social‐psychological isolation. Recent reviews have reinforced that these 14 interconnected hallmarks serve as core contributors to age‐related functional decline across multiple organ systems [35, 36, 37, 38, 39](Figure 1).

Precise quantification of aging biomarkers represents a critical step linking theory to the evaluation of anti‐aging interventions. In recent years, proteomic aging clocks have provided practical tools for this translation. The 2025 proteomic aging atlas constructed by Ding et al. offers the most comprehensive human aging map to date, using ultra‐high‐sensitivity mass spectrometry and machine learning to build proteomic clocks across 12 tissue types covering seven major physiological systems [40]. This study identified age 30 as the initial divergence point of aging trajectories, with adrenal tissue displaying the earliest features, and ages 45–55 as a critical turning point characterized by a surge in differentially expressed proteins. Furthermore, key circulating pro‐aging proteins were discovered, including GAS6, GPNMB, COMP, HTRA1, and IGFBP7, all of which directly induce vascular cell senescence; these findings shift aging research from intracellular mechanisms toward a systemic inter‐organ communication perspective. Complementarily, a 2024 study in ‘*Cell Metabolism’* applied machine learning to blood proteomics and identified protein signatures reflecting monocyte senescence and biological age‐encompassing frailty, inflammation, and metabolic dysfunction that robustly predict frailty and inflammatory states [11]. These advances capture the multisystem nature of aging networks and highly align with the ‘multi‐component, multi‐target, holistic regulation’ paradigm of TCM, thereby laying a foundation for the mechanistic elucidation and efficacy evaluation of traditional formulas.

### 3.3 Molecular Mapping of Core TCM Aging Theories Onto Hallmark Pathways

#### 3.3.1 Kidney Essence Depletion Theory

The ‘Kidney Essence Depletion Theory’ posits that aging is fundamentally driven by the depletion of the innate *kidney essence* reserve, which in turn leads to multisystem functional decline. At the molecular level, this process is manifested by a systemic reduction in the NAD^+^ pool [41] and the downregulation of Klotho expression [42, 43, 44], which involves biological markers such as genomic instability, telomere attrition, disabled macroautophagy, mitochondrial dysfunction, and stem cell exhaustion. NAD^+^ deficiency inhibits SIRT1‐mediated deacetylation repair and PARP1‐dependent DNA damage response, resulting in genome instability and accelerated shortening due to loss of heterochromatin in telomeric regions [45, 46, 47, 48, 49]; meanwhile, the decrease in NAD^+^ weakens the inhibitory effect of AMPK on mTORC1, hinders the initiation of autophagy by the ULK1 complex, and blocks the clearance of damaged organelles and protein aggregates [41, 46, 50, 51, 52, 53]. The decrease in Klotho protein disrupts the fine regulation of the Wnt/β‐catenin signal in the microenvironment of bone marrow mesenchymal stem cells (BMSCs), and impairs their self‐renewal and differentiation ability [54, 55, 56] (Table 1).

**TABLE 1:** Mapping core TCM aging theories to the 14 biological hallmarks of aging.

The classic theory of aging in TCM	Hallmarks of aging	Mechanisms	References
*Kidney essence depletion theory*	Genomic instability	Reduced NAD^+^ mediates the inhibition of PARP1 activity, which results in increased DNA damage	[46, 47, 50, 57]
	Telomere attrition	Reduced NAD^+^ affects the TRF2‐mediated telomere fusion process	[46, 48, 57]
	Epigenetic alterations	Reduced NAD^+^ inhibits SIRT1‐mediated deacetylation repair	[45, 46, 49]
	Disabled macroautophagy	NAD^+^ decreases and inhibits mTORC1‐AMPK‐ULK1	[46, 51, 52]
	Mitochondrial dysfunction	The decrease of NAD^+^ exacerbates mitochondrial RNA leakage and reduces mitochondrial function	[41, 46, 53]
	Stem cell exhaustion	Klotho protein reduction inhibits Wnt/β‐atenin signalling in stem cells	[54, 55, 56]
*Kidney deficiency and blood stasis theory*	Disabled macroautophagy	Overexpression of HIF‐1α impairs autophagy and lysosome function	[58, 59, 60]
	Mitochondrial dysfunction	HIF‐1α inhibits the activity of mitochondrial respiratory chain complex I/IV, leading to a decrease in ATP synthesis	[50, 61, 62]
	Chronic inflammation	Activate NLRP3 inflammasome	[63, 64, 65]
	Changes in the extracellular matrix	Hypoxia and inflammatory factors upregulate TGF‐β1/Smad signalling, disrupting MMPs/TIMPs balance	[66, 67, 68, 69]
*Qi‐blood theory*	Deregulated nutrient sensing	Elevated AMP/ATP ratio inhibits AMPK‐mTORC1 pathway	[70, 71, 72, 73]
	Mitochondrial dysfunction	Decreased mitochondrial quality and ATP level	[74, 75, 76]
	Cellular senescence	Inflammatory response mediates increased expression of p16INK4a, leading to G1 phase cell cycle arrest	[77, 78]
	Stem cell exhaustion	Exhaustion of haematopoietic and mesenchymal stem cells	[74, 79, 80]
	Altered intercellular communication	The decline in astrocyte function and glutamate release affects the functional interaction between neurons and astrocytes	[81, 82, 83]
	Psychosocial isolation	Inflammatory response mediates reduced metabolic flexibility and exacerbates social isolation	[84, 85, 86, 87]
*Spleen‐stomach theory*	Deregulated nutrient sensing	Disruption of intestinal epithelial tight junction proteins promotes the translocation of LPS into the bloodstream, leading to metabolic endotoxemia; Insufficient intake of leucine and short‐chain fatty acids	[88, 89, 90, 91]
	Loss of proteostasis	Leucine deficiency weakens the phosphorylation of 4E‐BP1/S6K1 and inhibits the synthesis of new proteins	[92, 93, 94]
	Mitochondrial dysfunction	Oxidative stress intensifies, SOD activity decreases, MDA content increases	[95, 96]
	Chronic inflammation	Systemic Th1/Th2 immune imbalance and bias toward Th2 polarization	[97, 98]
	Genomic Instability	Elevated 8‐hydroxydeoxyguanosine and aggravated mtDNA damage	[99, 100]
	Dysbiosis	Continuous compensatory activation of AMPK mediates downregulation of beneficial bacteria such as Bifidobacterium and Lactobacillus	[91, 101, 102]

#### 3.3.2 Kidney Deficiency and Blood Stasis Theory

Building upon the ‘Kidney Essence Depletion Theory’, the ‘Kidney Deficiency and Blood Stasis Theory’ extends this framework by emphasizing a positive feedback loop between *deficiency* and *stasis* [56, 103]. *Kidney deficiency* reduces NO bioavailability and increases ET‐1, leading to microvascular spasm and decreased erythrocyte deformability, resulting in chronic hypoxia and outer matrix sclerosis, characterized by chronic inflammation, extracellular matrix changes, and mitochondrial dysfunction. Hypoxia and accumulated damage‐associated molecular patterns activate the NLRP3 inflammasome, promote the release of IL‐1β/IL‐18, and maintain a low‐grade inflammatory state [61, 63, 64, 65]. Then hypoxia synergizes with inflammatory factors to upregulate TGF‐β1/Smad signalling, drive fibroblasts to transform into myofibroblasts, and break the balance of MMPs/TIMPs, resulting in abnormal cross‐linking of collagen and extracellular matrix sclerosis [66, 67, 68, 69]. In addition, hypoxia regulator HIF‐1α inhibits mitochondrial respiratory chain complex I/IV activity, resulting in decreased ATP synthesis [61, 62, 69], increased lysosomal membrane permeability, hindered autophagosome lysosome fusion, and exacerbated impaired autophagic flux [58, 59] (Table 1).

#### 3.3.3 Qi‐Blood Theory

*Qi* and *blood* constitute a functional energy‐material couple, wherein *qi* corresponds to bioenergetic flux and signalling cascade activity, while *blood* represents oxygen delivery and nutrient substrate supply. During aging, the transition of *qi* and *blood* from abundance to *deficiency* drives an imbalance between energy production and metabolic demand, which intersects with multiple hallmarks of aging, including dysregulated intercellular communication, mitochondrial dysfunction, stem cell exhaustion, cellular senescence, impaired nutrient sensing, and even psychosocial isolation. *Qi deficiency* caused the increase of AMP/ATP ratio and a decrease of Insulin/IGF‐1 signal sensitivity, disturbs the AMPK‐mTORC1 nutrient sensing axis, and destabilizes the cell between synthesis/catabolism [70, 71, 72, 104]; *blood deficiency* leads to insufficient perfusion, reducing the oxygenation microenvironment and substrate supply required by haematopoietic and mesenchymal stem cells [74, 79, 80]. *Qi stagnation* or *qi deficiency* causes poor blood flow, hindering neurotransmitter and cytokine transmission and energy supply between neurons and glial cells [81, 82]. Long‐term *deficiency* of *qi* and *blood* activates Rb/p16INK4a pathway [77, 78], induces irreversible cycle arrest of endothelial and parenchymal cells, and leads to a vicious cycle of decreased central metabolic flexibility and social function withdrawal [84, 85, 86, 87] (Table 1).

#### 3.3.4 Spleen‐Stomach Theory

The spleen and stomach are regarded as the ‘postnatal foundation’ in TCM, responsible for nutrient transformation and distribution, as well as intestinal excretion. With aging, their functional decline, manifested by small intestinal villous atrophy and gut dysbiosis, impairs the generation and transport of *qi* and *blood*, thereby accelerating multi‐organ senescence. This pathological trajectory converges with several canonical hallmarks of aging, including disrupted nutrient sensing, loss of proteostasis, mitochondrial dysfunction, chronic inflammation, and DNA damage. The abnormal transportation of spleen and stomach destroys the intestinal epithelial tight junction, promotes the translocation of LPS through the portal vein, activates the TLR4/MyD88 pathway in the liver, and induces metabolic endotoxemia [88, 89, 90, 91]; at the same time, the lack of substrate causes continuous compensatory activation of AMPK, excessive inhibition of anabolism, and dysbiosis of the constituent flora, resulting in insufficient uptake of leucine and short chain fatty acids [91, 101]. Leucine deficiency impairs 4E‐BP1/S6K1 phosphorylation downstream of mTORC1, inhibits nascent protein synthesis [92, 93, 94], while limiting mitochondrial beta‐oxidation and TCA cycle flux, reducing respiratory chain complex enzyme activity and accumulating mtDNA mutations [95, 99, 100] (Table 1).

## 4 Representative TCM Formulas in Modulating Aging Hallmarks: Mechanisms and Evidence

Chinese herbal formulas have been shown to possess multi‐targeting potential against aging hallmarks. In this section, representative formulas are categorized into four groups according to their predominant mechanisms: (1) modulators of mitochondrial homeostasis and autophagy; (2) modulators of cellular senescence and the inflammatory microenvironment; (3) modulators of stem cell maintenance and epigenetic reprogramming; and (4) modulators of gut microecology and systemic metabolism. This classification is intended to highlight the mechanistic focus of each formula category on specific molecular nodes, rather than to simplistically confine their actions. Each formula inherently functions as a multi‐target network regulator; thus, while the primary mechanism is emphasized, the broader multi‐target regulatory profile is also delineated within each subsection. Collectively, this framework provides critical support for the modern scientific interpretation of the holistic anti‐aging effects of Chinese herbal formulas (Figure 2 and Table 2).

**FIGURE 2:**
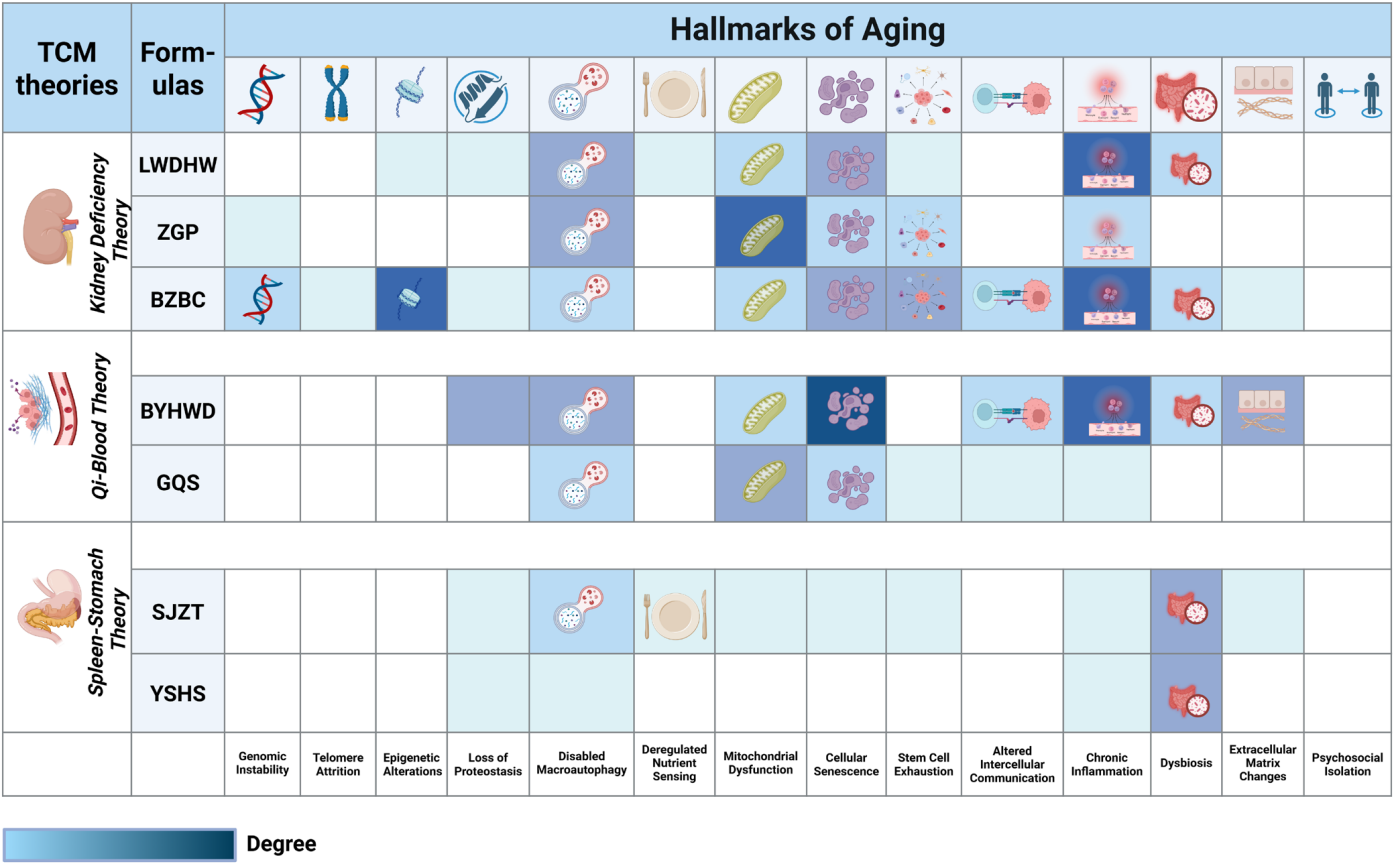
Regulatory effects of TCM formulas on the 14 hallmarks of aging stratified by therapeutic principles. This heatmap illustrates the intervention effects of seven classic TCM formulas belonging to three therapeutic categories on 14 hallmarks of aging. The colour gradient from light to dark blue indicates the increasing number of supporting studies for each formula‐hallmark regulatory relationship.

**TABLE 2:** Representative TCM formulas modulate the hallmarks and mechanisms of aging.

Hallmarks of aging	Representative TCM formulas	Mechanisms	References
Genomic instability	ZGP and YGP	Inhibit DNA damage signals mediated by γ‐H2AX and CHEK2, and promote DNA damage repair	[105]
	BZBC	Inhibition of DNA damage marker gamma H2AX accumulation	[15]
Telomere attrition	BZBC	In a randomized double‐blind trial, it can activate telomerase	[16]
	Eight‐treasure soup	Upregulation of DNA helicase expression facilitates G‐quadruplex and telomere maintenance	[106]
Epigenetic alterations	LWDHW	Inhibition of DNMT1 expression and ER‐α gene methylation	[107]
	BZBC	Regulating DNA methylation levels; Regulating DHRS2 deacetylation and eNOS Thr495 phosphorylation	[18, 19]
	GEG	Regulating histone methylation levels in the promoter region of the p16Ink4a gene	[108]
Loss of proteostasis	LWDHW	Activate the YAP‐autophagy axis	[13]
	BZBC	Inhibition of protein kinase Cδ	[19]
	BYHWD	Regulating TLR4/NF‐KB/NLRP3, AMPK, gut microbiota‐C/EBP β/AEP signalling pathways	[109, 110]
	SJZD	Inhibition of PI3K‐AKT and P38 MAPK signalling pathways	[111]
	KXS	Regulating the NLRP3/Caspase‐1 signalling pathway to target Aβ accumulation; Heat shock response and autophagy, clearing PolyQ aggregates	[112, 113, 114]
	Huangjing Yangji	Activate the SIRT1/ATF5 mediated UPRmt pathway	[115]
	Yuan Zhi Powder	Reduce phosphorylated Tau protein aggregation	[116]
Disabled macroautophagy	LWDHW	Activate YAP autophagy axis; Downregulate the expression of ATG5, Beclin1, and LC3B; Regulating the SIRT1/PI3K/AKT pathway to regulate autophagy	[12, 13, 14]
	ZGP and YGP	Regulating PINK1/Parkin and BEC‐1/PINK‐1 dependent mitochondrial phagocytosis	[117, 118]
	BZBC	Promote the expression of autophagic protein Beclin1 and LC3‐II transformation	[17, 119]
	GEG	Regulating AMPK/mTOR/ULK1 and PINK1/Parkin pathways	[120, 121]
	YSHSG	Mediating the mTOR/AMPK/PI3K/Akt pathway	[122]
	BYHWD	Activate the SIRT1 pathway, inhibit miR‐665 to promote DRAM1 protein expression	[109, 123]
	SJZD	Promote the restoration of the bidirectional autophagy balance of positively correlated metabolites in the microbiota	[124, 125]
	KXS	Two key arms of the regulating protein quality control system	[114]
	Bufei Yishen Formula	Activate AMPK‐Sirt1‐FoxO3a pathway	[126]
	Wuzi Yanzong Pill	Regulate the AKT/mTOR and mTORC1‐mTORC2 signalling pathways	[127]
	Luo Zhi Tong decoction	Regulating the AMPK/mTOR signalling pathway	[128]
	Ginseng tonifying decoction	Upregulation of LC3 and Beclin‐1	[129]
Deregulated nutrient sensing	LWDHW	Regulating the cAMP/PKA/CREB signalling pathway	[130]
	BYHWD	Promote disaccharide synthesis	[131]
	KXS	APP/PS1 regulates SIRT3 mediated neuronal apoptosis	[132, 133]
	Rehmannia decoction	Upregulation of INSR, IRS1, IRS2, IGF1 expression, downregulation of IGFBP3	[134]
	Liujunzi decoction	Regulate the IIS/mTOR signalling pathway	[135]
Mitochondrial dysfunction	ZGP and YGP	Inhibiting Drp1‐mediated mitochondrial fission and activating AMPK/PGC‐1 α—driven mitochondrial biosynthesis; Upregulation of Nrf2, HO‐1, and GPX4, downregulation of ACSL4; Restoring the expression of PGC‐1 α and TFAM	[136, 137]
	BZBC	Stabilize mitochondrial membrane potential and alleviate oxidative stress and aging in endothelial cells	[17, 119]
	GEG	Enhance the activity of mitochondrial function‐related proteins Complex I‐V	[120, 121]
	BYHWD	Regulate the SIRT1/AMPK signalling pathway to enhance glucose metabolism and TCA cycle function; Activate the AMPK/Drp1 pathway to improve mitochondrial dynamics disorder	[138, 139]
	SJZD	Reduce ROS generation and oxidative stress, and increase mitochondrial membrane potential	[140]
	KXS	Adjust the APP/PS1, SIRT1/FSP1 pathway	[112, 132, 141]
	Bushen Huoxue Recipe	Regulate mitochondrial respiratory chain complex and ROS‐SOD	[142]
	Huangjing Yangji Decoction	Improve particle morphology, membrane potential, and ATP generation	[115]
	Jinkui Shenqi Wan	Improve energy metabolism and reduce oxidative damage	[139]
	Tongqiao Huoxue Decoction	Restore ATP production, stabilize membrane potential, and reduce superoxide generation	[129]
	Liujunzi Decoction	Regulate the p16/p21 pathway to improve mitochondrial function	[135]
	Jianpi Yiqi Decoction	Activate the Pink1/Parkin pathway	[143]
Cellular senescence	LWDHW	Reduce the expression of p53 and p21 proteins and improve the cell cycle; Regulate the JPX‐STING‐IRF3 pathway	[13, 144]
	ZGP and YGP	Promote DNA damage repair and reduce SASP; regulate the Notch1/Nrf2 pathway, optimizing the cell cycle	[105, 137, 145]
	BZBC	Reduce SA‐β‐gal activity, P16/P21 expression	[15, 16, 17]
	GEG	Regulate cell survival rate, cell cycle, and SA‐β‐gal activity	[108]
	BYHWD	Activation of the SIRT1 pathway inhibits H_2_O_2_‐induced cellular senescence	[146]
	SJZD	Inhibit the expression of p53, p‐p53, and p21, and downregulate p38 phosphorylation	[140]
	KXS	Reduce NLRP3, ASC, Caspase‐1, and GSDMD	[113]
	Eight‐treasure soup	Regulating the C1qbp‐cGAS‐STING pathway‐mediated cellular aging	[147]
	Jianpi Qingre Huoxue	Regulating the STAG1/TP53/P21 signal axis	[148]
Stem cell exhaustion	LWDHW	Activate the YAP‐autophagy axis to alleviate the aging of BMSCs	[13]
	ZGP and YGP	Increase BMSCs osteogenic differentiation transcription factors Runx2, COL‐1, ALP, OPN; Regulate Notch1/Nrf2 to maintain OSC dryness	[105, 137, 145, 149]
	BZBC	Upregulation of the expression of COL17A1, OCT4, SOX2, NANOG	[150]
	GEG	Delaying HSCs aging	[108, 151]
	SJZD	Upregulation of stem cell markers	[140]
Altered intercellular communication	BZBC	Inhibit the abnormal polarization of microglia; Inhibit NLRP3 inflammasome‐mediated cell apoptosis	[17, 152]
	GEG	Promote the SLAM‐SAP pathway, mediate Tbet production in T cells, and reduce IFN‐γproduction	[151]
	BYHWD	Inhibition of miR‐665 promotes DRAM1 protein expression Regulating extracellular vesicle miR‐590‐5p to downregulate SLC8A3 expression	[123, 153]
Chronic inflammation	LWDHW	Regulating the AMPK/SIRT1/NF‐κB pathway	[154]
	ZGP and YGP	Raise IL‐10 levels and reduce MIF and TNF‐α levels	[155]
	BZBC	Inhibit the TNF‐α/NF‐κB, TLR4/NFκB, and KEAP1‐NRF2 pathways Inhibit NLRP3 inflammasome‐mediated cell apoptosis Inhibit the abnormal polarization of microglia	[15, 17, 119, 150, 152]
	GEG	Promote the SLAM‐SAP pathway, regulate T‐bet expression in T cells, and reduce IFN‐γ production	[151]
	YSHSG	Upregulation of miR‐339‐5p targeting inhibition of TGF‐β1/Smad pathway	[156]
	BYHWD	Inhibit the inflammatory pathways of TLR4/NF‐κB/NLRP3, CaM/CaMKII/MAPK, p53/cGAS/STING inflammatory axis M1/M2 polarization reprogramming of macrophages mediated by miR‐590‐5p Inhibition of vascular inflammation through downregulation of SLC8A3 mediated by extracellular vesicle miR‐590‐5p	[109, 110, 153, 157, 158]
	SJZD	Regulate the TNF‐α/NF‐κB pathway	[125]
	KXS	Promote mitochondrial autophagy	[113, 132]
	Tongqiao Huoxue Decoction	Inhibition of NLRP3 inflammasome activation	[129]
	Danggui Buxue Decoction	Improve T cell immune dysfunction	[159]
Dysbiosis	LWDHW	Regulating the TMA‐trimethylamine oxide metabolic axis dependent on gut microbiota Increase the relative abundance of beneficial bacteria (such as lactobacilli), and reduce the abundance of harmful bacteria	[160, 161, 162]
	BZBC	Inhibiting NLRP3 inflammasome‐mediated cell apoptosis to restore the ecological imbalance of gut microbiota and increase the abundance of beneficial bacteria	[15, 17]
	YSHSG	Mediating the regulation of gut microbiota through the ‘gut‐kidney’ axis	[163]
	BYHWD	Regulating gut microbiota‐C/SJZD/AEP, regulating the abundance of probiotics/harmful bacteria	[153, 164]
	SJZD	Improve intestinal permeability and promote the generation of positively correlated metabolites in the microbiota	[124]
	KXS	Reshaping gut microbiota and its metabolites	[133]
	Suanzaoren	Affects the metabolic pathways of tryptophan, alanine, aspartate, and glutamate	[165]
Changes in the extracellular matrix	BYHWD	Regulating extracellular vesicle miR‐590‐5p to downregulate SLC8A3 expression	[123, 153]
Psychosocial Isolatio	Niuhuang Qingxin Wan	Improve the low activation state of the prefrontal cortex	[166, 167]

### 4.1 Mitochondrial Homeostasis and Autophagy Modulators

Mitochondrial dysfunction and impaired autophagy form a core cellular quality‐control axis in aging [168, 169]. Declining oxidative phosphorylation and autophagic flux create a vicious cycle of ROS accumulation and organelle damage, underpinning neurodegeneration and metabolic disorders [170]. Thus, restoring mitochondrial homeostasis and autophagic balance represents a fundamental strategy to counteract aging at its bioenergetic roots.

#### 4.1.1 Zuogui Pill (ZGP) and Yougui Pill (YGP)

ZGP and YGP are both classic *kidney tonifying* prescriptions, which have the effects of nourishing *kidney yin* and warming *kidney yang*, respectively. In *Caenorhabditis elegans (C. elegans)*, ZGP and YGP extend healthspan by activating PINK1/Parkin‐mediated mitophagy [117]. Extending from systemic aging to organ‐specific effects: in the nervous system, ZGP ameliorates memory deficits in an Alzheimer's disease (AD) mouse model by inhibiting Drp1‐mediated mitochondrial fission and activating AMPK/PGC‐1α‐driven mitochondrial biogenesis [136]. In the reproductive system, ZGP reduces oxidative damage in ovarian and testicular cells, restores mitochondrial membrane potential, and couples mitophagy with anti‐apoptotic effects [105, 137, 145]. In the skeletal system, YGP promotes chondrocytic autophagic clearance, thereby preserving joint integrity [149, 171]. Beyond this core action on mitochondria, ZGP also maintains stemness of ovarian and bone marrow‐derived mesenchymal stem cells via Notch1/Nrf2 and SIRT1 pathways, while reducing the SASP and DNA damage [105, 137, 145]. Collectively, these findings establish a multi‐tiered regulatory system centred on mitochondrial functional remodelling, complemented by the modulation of stem cell exhaustion, cellular senescence, chronic inflammation, epigenetic alterations, and genomic instability.

#### 4.1.2 Kaixin San (KXS)

KXS targets neuronal mitochondrial homeostasis and modulates the peripheral gut–brain axis to counteract systemic aging. Within the brain, KXS activates the PINK1/Parkin axis to sustain autophagic flux and clear damaged mitochondria in AD mice [112, 113, 132, 172]. In polyQ transgenic *C. elegans* , KXS enhances heat shock response and autophagy to eliminate polyQ aggregates, thereby preserving both mitochondrial function and proteostasis [114]. Focusing on neuronal integrity, its ginsenoside components provide metabolic support via DHFR‐related folate metabolism, which upregulates SIRT3 and enhances neuronal activity [112, 132]. Concurrently, KXS attenuates neuroinflammation and inhibits ferroptosis through modulation of the NLRP3/caspase‐1/GSDMD and SIRT1/FSP1 pathways [113, 141, 172, 173, 174]. Beyond its direct cerebral effects, KXS also regulates interorgan crosstalk: in AD patients, it restores intestinal homeostasis by modulating gut microbiota composition and metabolite abundance, thereby ameliorating cognitive impairment [133]. Collectively, these findings establish a mitochondrial autophagy‐centred multi‐target neuroprotective strategy against aging, with KXS targeting multiple aging hallmarks including mitochondrial dysfunction, loss of proteostasis, chronic inflammation, and dysbiosis.

#### 4.1.3 GQS

GQS originated from the Ming‐Dynasty formula *Guilu Erxian Jiao* recorded in ‘Yi Fang Kao’, and was later refined by gradually adding herbs such as Astragalus and Salvia based on Zhang Jingyue's *kidney tonifying* prescriptions, ultimately finalizing as a modern tonic compound that replenishes *qi* and *blood*, harmonizes *yin* and *yang*, tonifies both spleen and kidney, and supplements without causing stagnation. In a clinical study focusing on chronic fatigue syndrome, a condition characterized by mitochondrial dysfunction, chronic inflammation, and cellular senescence, GQS achieved a markedly effective rate of 55.56% [20, 21]. Mechanistically, GQS regulates intestinal oxidative stress, thereby restoring the homeostatic balance of intestinal stem cell proliferation and differentiation, which in turn preserves intestinal barrier integrity and absorptive function, ultimately contributing to healthspan extension. At the component level, ginseng acidic polysaccharide WGPA‐A has been identified as a key substance that ameliorates chronic fatigue syndrome; it significantly reverses degenerative changes in the mitochondrial structure of striated muscle, while reducing MDA and LDH levels and increasing SOD and GPX activities [175]. Meanwhile, ginsenoside Rg1 induces microglial mitophagy through the PINK1/Parkin signalling pathway, thereby inhibiting NLRP3 inflammasome activation [176]. Another key substance, astragaloside IV, activates AMPK signalling and improves mitochondrial function [177, 178, 179]. Collectively, these findings suggest that GQS targets multiple aging hallmarks, including mitochondrial dysfunction, chronic inflammation, and impaired macroautophagy, highlighting its potential value for further anti‐aging research and product development.

### 4.2 Cellular Senescence and Inflammatory Microenvironment Modulators

Cellular senescence and chronic inflammation serve as central amplifiers in the aging process, wherein senescent cells secrete SASP factors that drive chronic inflammation, disrupt tissue homeostasis, and mediate the development of age‐related atherosclerosis, neurodegenerative diseases, and metabolic disorders [180, 181, 182]. Targeting it breaks the vicious cycle of senescent cell accumulation and sustained inflammation—a critical strategy for preserving tissue function.

#### 4.2.1 BYHWD

BYHWD is a classic formula widely used for cerebrovascular and cardiovascular diseases. It targets the SASP‐inflammation axis, with its cardiovascular anti‐aging effects primarily mediated through SIRT1 activation, which suppresses SASP factors (IL‐1β, TNF‐α, IL‐6) and downregulates p16 and p21 expression. Mechanistically, BYHWD modulates multiple inflammatory pathways, including TLR4/NF‐KB/NLRP3, CaM/CaMKII/MAPK, and p53/cGAS/STING cascades [138, 153, 157, 158, 183]. Notably, BYHWD uniquely mediates exosomal miR‐590‐5p targeting SLC8A3 to reprogram macrophage polarization from M1 to M2 phenotype, thereby remodelling the vascular immune microenvironment [172]. Beyond its core action on the SASP‐inflammation axis, BYHWD improves mitochondrial dynamics via the AMPK/Drp1 pathway [146, 184, 185], enhances cerebral glucose metabolism and TCA cycle flux through SIRT1/AMPK activation [138, 153, 157, 183], restores extracellular matrix homeostasis by normalizing the MMP‐9/TIMP‐1 balance [160], and ameliorates cognitive function through the gut microbiota/EBPβ/AEP axis. Collectively, these findings establish an eight‐dimensional regulatory network targeting chronic inflammation, cellular senescence, autophagy dysfunction, mitochondrial damage, dysbiosis, protease inactivation, intercellular communication disruption, and extracellular matrix damage.

#### 4.2.2 Liuwei Dihuang Wan (LWDHW)

Acting as a classic *Kidney Yin‐nourishing* formula, LWDHW combats aging through suppression of cellular senescence and chronic inflammation, a process centred on the kidney and radiating outward to multiple remote organs. In the reproductive system, LWDHW alleviates ovarian damage via the SIRT1/PI3K/AKT signalling pathway [12] and reduces testicular inflammation through the AMPK/SIRT1/NF‐KB axis [154]. In the skeletal system, it activates the YAP‐autophagy axis in BMSCs, reduces p53/p21 expression, and promotes osteogenic differentiation, thereby counteracting age‐related bone loss [13]. In the brain, LWDHW inhibits microglial inflammation and modulates the cAMP/PKA/CREB pathway to alleviate cognitive impairment [130]. In the vasculature, it protects against endothelial dysfunction by inhibiting DNMT1‐mediated ERα methylation, concurrently regulating epigenetic alterations and cellular senescence [107]. In terms of autophagy regulation, LWDHW upregulates ATG5, Beclin1, and LC3B via the PI3K/Akt/FoxO3a pathway to ameliorate macroautophagy dysfunction [14]. In terms of microecological remodelling, it restores gut dysbiosis (increasing *Lactobacillus* and reducing *Proteobacteria*), thereby peripherally modulating reproductive aging [160]. Collectively, LWDHW constructs a systemic anti‐aging network centred on cellular senescence and chronic inflammation, while secondarily targeting macroautophagy dysfunction, epigenetic alterations, stem cell exhaustion, dysbiosis, and protease inactivation.

### 4.3 Epigenetic Reprogramming and Stem Cell Maintenance Modulators

Epigenetic drift and stem cell exhaustion jointly erode regenerative capacity with age. Dysregulated DNA methylation, histone modifications, and silenced stemness genes impair self‐renewal, while aging exacerbates these changes [186, 187, 188]. Targeting this group of aging hallmarks complements energy maintenance and inflammatory‐immune modulation strategies through the restoration of cellular stemness and tissue repair potential.

#### 4.3.1 BZBC

BZBC is a modern formula that integrates multiple anti‐aging principles and provides compelling evidence for epigenetic remodelling. In a randomized trial involving 530 healthy adults, BZBC increased telomerase activity by 76.7%, equivalent to a five‐year reversal of physiological age [16]; in aged mice, it reversed DNA methylation age and upregulated skin stemness markers including OCT4, SOX2, NANOG, and COL17A1 [18, 119]. Mechanistically, BZBC links epigenetic regulation to mitochondrial function through SIRT3‐dependent deacetylation of DHRS2 [19]. Beyond its core epigenetic effects, BZBC suppresses NLRP3 inflammasome activation and reduces p16/p21 expression [17, 189], and modulates the gut microbiota‐spermidine axis (enriching *Akkermansia muciniphila* ) while inhibiting microglial inflammatory polarization, thereby reversing SASP‐driven intercellular communication deficits [152, 190]. Collectively, BZBC represents a multi‐system, multi‐target intervention strategy that bridges fundamental mechanisms with clinical evidence.

#### 4.3.2 Guilu Erxian Glue (GEG)

GEG is renowned for replenishing essence and benefiting marrow, and in modern research it directly targets haematopoietic stem cells (HSCs) aging. It inhibits p16 expression through histone methylation at the p16Ink4a promoter, thereby maintaining HSC self‐renewal, while also modulating the SLAM‐SAP pathway to control T‐cell IFN‐γ production and alleviate immune‐mediated HSC damage [108, 151]. Beyond the haematopoietic system, GEG restores autophagic flux and improves mitochondrial function in chondrocytes via the AMPK/mTOR/ULK1 and PINK1/Parkin pathways [120, 121]. Collectively, by targeting six hallmarks of aging—stem cell exhaustion, mitochondrial dysfunction, impaired autophagy, dysregulated intercellular communication, inflammatory response, and cellular senescence—GEG establishes a direct link between the TCM concept of ‘*essence*’ (*Jing‐sui*) and the regulation of stem cell aging.

#### 4.3.3 Gut Microecology and Systemic Metabolism Modulators

Age‐related gut dysbiosis impairs barrier integrity, promotes endotoxin translocation, and sustains low‐grade systemic inflammation through the gut‐organ axis, thereby integrating and exacerbating the pathological effects of other aging hallmarks [191, 192]. This ‘peripheral’ regulatory strategy complements intrinsic direct intervention approaches by targeting external drivers originating from the gut environment.

#### 4.3.4 Sijunzi Decoction (SJZD)

SJZD is a classic formula for invigorating the spleen and supplementing *qi*. It delays aging by modulating the gut microbiota and restoring intestinal barrier function. In the intestines of aged rats, SJZD significantly restores gut homeostasis by increasing the levels of beneficial bacteria (such as *Lactobacillus* and *Bifidobacterium*) and short‐chain fatty acids, thereby alleviating systemic inflammation and oxidative stress [124, 125]. Concurrently, in the brain, SJZD upregulates the expression of key autophagy proteins Beclin‐1 and LC3‐II, thus protecting hippocampal neurons from inflammation and aging‐related damage [125]. Beyond the gut and brain, SJZD prevents skin aging by reducing UVB‐induced epidermal thickening, increasing collagen and elastic fibres, lowering oxidative stress markers (MDA, ROS), and upregulating antioxidant enzymes (CAT, T‐SOD) and stemness markers [140]. Furthermore, it alleviates age‐related osteoporosis and hair loss by inhibiting the PI3K‐AKT and p38 MAPK pathways [111]. Collectively, SJZD targets multiple aging hallmarks, including dysbiosis, macroautophagy impairment, cellular senescence, and stem cell exhaustion, supporting the TCM concepts of the ‘spleen‐muscle’ and ‘spleen‐intellect’ connections (Table 2).

#### 4.3.5 Yishen Huashi Granule (YSHSG)

Derived from Li Dongyuan's Shengyang Yiwei Tang, YSHSG targets gut‐kidney axis. In diabetic kidney disease models, YSHSG restores gut dysbiosis (increasing *Lactobacillus*, reducing the Firmicutes/Bacteroidetes ratio), lowers intestinal permeability, and reduces uremic toxins (indoxyl sulfate), thereby acting on the gut‐kidney axis to delay renal aging and fibrosis [163]. To counteract chronic inflammation, YSHSG upregulates miR‐339‐5p to inhibit the TGF‐β1/Smad pathway, alleviating renal fibrosis [185]. Furthermore, YSHSG improves mitophagy and modulates the mTOR/AMPK/PI3K/Akt signalling pathways to protect against mitochondrial dysfunction, thereby mitigating organ damage [122]. Collectively, YSHSG provides a multi‐mechanistic approach to delay renal aging by targeting multiple aging hallmarks (Table 2).

## 5 Core Bioactive Constituents and Their Anti‐Aging Mechanisms

### 5.1 Major Compound Classes

The anti‐aging effect of TCM formulas is mediated by various bioactive ingredients, which can be roughly divided into several chemical categories. Among them, saponins, flavonoids and their glycosides, polysaccharides, organic acids, and other small molecules are the most widely studied categories. These compound categories together constitute the multi‐target and multi‐level anti‐aging mechanism of TCM formulas, laying the chemical foundation for the representative activity of TCM formulas described in the previous text.

### 5.2 Representative Active Compounds

#### 5.2.1 Saponins

Saponins are a type of amphiphilic molecule composed of hydrophobic aglycones and hydrophilic sugar chains connected by glycosidic bonds. This unique ‘lipophilic–hydrophilic’ structure allows them to embed into cell membranes, bind to membrane cholesterol, or regulate membrane protein functions. As the most extensively studied and well‐evidenced anti‐aging ingredient in traditional prescriptions, it has effects ranging from precise cell regulation to organ functional protection. For instance, ginsenosides Rg1 and Rb1, which are abundant in KXS and SJZD [193, 194, 195], activate the AMPK/mTOR and AMPK/SIRT1 signalling axes, enhancing mitophagy and mitochondrial function [196, 197, 198, 199, 200]. At the organ level, ginsenosides exhibit systemic anti‐aging potential in multiple organs including the brain, heart, kidneys, testes, and muscles [199, 201, 202]. Astragaloside IV, a key saponin in BYHWD and GQS [203], activates Parkin‐mediated autophagy to maintain mitochondrial quality and functional homeostasis, thereby counteracting oxidative stress [11, 204]. In terms of stem cell homeostasis, astragaloside IV improves mitochondrial function in HSCs via the PGC‐1α pathway, while also targeting ketohexokinase to restore intestinal stem cell proliferation and homeostasis, thereby delaying vascular‐intestinal aging [205, 206]. Notoginsenoside R1 in modified formulas inhibits NLRP3 inflammasome activation and reduces SASP factor levels [207, 208]. In summary, saponins precisely target nutritional perception disorders, autophagy disorders, and mitochondrial damage, forming the core molecular cornerstone of TCM anti‐aging.

#### 5.2.2 Flavonoids and Their Glycosides

Flavonoids have a carbon skeleton of C6‐C3‐C6, usually existing in the form of free glycosides or glycosides synthesized with sugars. Their polyphenolic structure endows them with strong electron supply ability and metal chelating activity, thereby efficiently scavenging free radicals and regulating redox signals. Their effects span three biological levels—subcellular, cellular, and organ—manifested as mitochondrial quality control, suppression of the cellular senescence phenotype, and integration of organ functions, complementing saponin compounds in mechanism. At the subcellular level, kaempferol from ZGP and YGP activates the PINK1/Parkin pathway to induce mitophagy, thereby extending healthspan in *C. elegans* and balancing the osteogenic versus adipogenic differentiation of BMSCs, alleviating postmenopausal osteoporosis [209, 210]. At the cellular phenotype level, quercetin from BYHWD suppresses the SASP, reduces p16/p21 expression, and activates SIRT1, thereby counteracting vascular and cardiac aging [211]. Finally, icariin, a prenylated flavonoid from BZBC, alleviates skin photoaging by activating the KEAP1‐NRF2 axis and protects vascular endothelium via the PI3K/Akt/eNOS pathway, demonstrating a multi‐organ integrated anti‐aging effect [212, 213].

#### 5.2.3 Polysaccharides

Polysaccharides are high molecular weight polymers composed of more than 10 monosaccharides connected by glycosidic bonds. They have a large molecular weight and complex structure, making them difficult to digest and absorb directly. However, they can be selectively fermented and utilized by gut microbiota to produce active metabolites such as short‐chain fatty acids. Different from directly targeting single‐cell antioxidants, polysaccharides tend to regulate systemic aging markers such as gut microbiota, immune aging, and stem cell exhaustion, and have peripheral anti‐aging effects [214]. Focusing on stem cell exhaustion, *Astragalus* polysaccharide, the main component of BYHWD, enhances the proliferation of umbilical cord‐derived mesenchymal stem cells and modulates the immune network both in vitro and in vivo [215]. *Angelica sinensis* polysaccharide from Si Wu Tang protects neural stem cells and preserves the function of haematopoietic stem/progenitor cells in aged mice, thereby alleviating age‐related immune decline [216]. Notably, *Panax ginseng* polysaccharide from SJZD remodels the gut microbiota composition, enriches beneficial fatty acid metabolism, and activates AMPK/SIRT1 signalling to suppress chronic low‐grade inflammation [217]. Furthermore, *Rehmannia glutinosa* polysaccharide (RGP70‐1) from LWDHW promotes DAF‐16 nuclear translocation and enhances antioxidant and anti‐inflammatory defences [218]. Collectively, these polysaccharides reverse inflammaging and maintain tissue homeostasis, making them an indispensable systemic regulator in the network of TCM anti‐aging drugs.

#### 5.2.4 Organic Acids and Other Small Molecules

Organic acids and other small molecules usually contain carboxyl or phenolic hydroxyl groups, alcohol hydroxyl groups, with a small molecular weight and high polarity. They are easy to penetrate cell membranes and directly participate in core metabolic pathways such as the TCA cycle and fatty acid oxidation. Despite their low content in TCM extracts, they fill the functional blind spots of other components in metabolic inflammation interference and mitochondrial homeostasis. Ferulic acid activates Nrf2 and suppresses NF‐κB, thereby reducing cognitive decline [219]. Moreover, it promotes mitophagy via the PINK1/Parkin pathway and upregulates SIRT1, thereby delaying cardiac aging [220]. Chlorogenic acid (Yin Qiao San) alleviates gut dysbiosis‐induced inflammaging by inhibiting NLRP3 activation [221, 222]. Tanshinone IIA exerts neuroprotective effects in aged POCD rat models by activating the Nrf2/SLC7A11/GPX4 axis, reducing the expression of hippocampal inflammatory factors, and inhibiting ferroptosis [223]. Collectively, these small molecules target chronic inflammation, mitophagy, and cellular senescence, thereby enhancing the multi‐target synergistic effect of TCM formulations.

### 5.3 Multi‐Compound Synergy

The anti‐aging efficacy of TCM formulas arises from synergistic interactions among multiple constituents rather than the isolated effects of single compounds. These synergies can be categorized into three modes: pharmacokinetic synergy, pharmacodynamic synergy, and toxicity‐modulating synergy (Figure 3).

**FIGURE 3:**
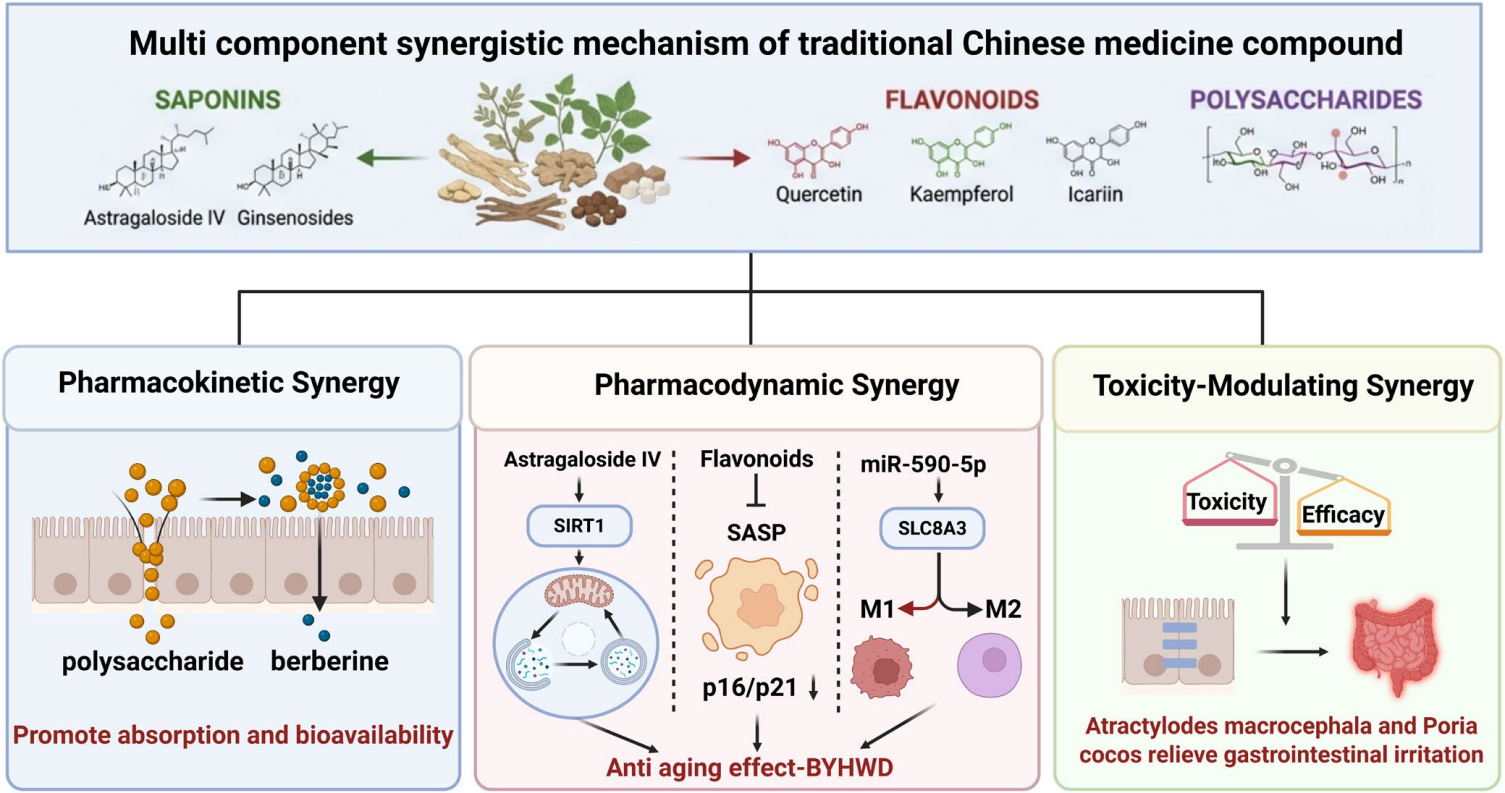
Multi‐component synergistic mechanism of TCM compound. TCM compounds exert holistic therapeutic effects through the synergistic actions of multiple bioactive components, primarily saponins, flavonoids, and polysaccharides. This synergy manifests in three core dimensions: (1) Pharmacokinetic synergy, where polysaccharides enhance the intestinal absorption and bioavailability of co‐administered components such as berberine; (2) Pharmacodynamic synergy, exemplified by BYHWD, in which distinct active components target complementary anti‐aging pathways: Astragaloside IV activates the SIRT1‐mediated mitochondrial regulation pathway, flavonoids inhibit the SASP and downregulate senescence markers p16/p21, and miR‐590‐5p modulates macrophage polarization from the pro‐inflammatory M1 to the anti‐inflammatory M2 phenotype via targeting SLC8A3; (3) Toxicity‐modulating synergy, where herbs including *Atractylodes macrocephala* and *Poria cocos* alleviate gastrointestinal irritation induced by other components, thereby balancing therapeutic efficacy and systemic toxicity.

#### 5.3.1 Pharmacokinetic Synergy

Pharmacokinetic synergy enhances bioavailability by modulating the absorption behaviour of key constituents. In Coptis chinensis decoction, polysaccharide‐based particle aggregates formed during decoction significantly influence the intestinal absorption of berberine, a poorly water‐soluble component, by increasing its solubility and permeability [224]. In the Jinshui Chenfei formula, iterative functional screening identified a combination of isoliquiritin, glycyrrhizic acid, and gallic acid. At specific concentration ratios, this triple combination synergistically suppresses the mRNA expression of TNF‐α, IL‐1β, IL‐6, CD206, and Arg‐1 in macrophages, with effects markedly superior to those of individual components [225]. Such synergistic interactions facilitate more efficient transmembrane transport of active compounds, thereby increasing their systemic exposure.

#### 5.3.2 Pharmacodynamic Synergy

Pharmacodynamic synergy enables formulas to cover the aging regulatory network in a multi‐target manner. In BYHWD, astragaloside IV induces SIRT1‐mediated autophagy to maintain mitochondrial homeostasis; coexisting flavonoids such as quercetin suppress the SASP and reduce p16/p21 expression; meanwhile, formula‐derived exosomes carrying miR‐590‐5p target SLC8A3 to reprogram M1/M2 macrophage polarization and alleviate vascular inflammation [226, 227, 228]. These three mechanisms act on cellular autophagy, cellular senescence phenotype, and immune microenvironment, respectively, and thus jointly delay vascular aging through distinct pathways. This multi‐target, multi‐level mode of action matches the multifactorial nature of aging networks and embodies the therapeutic advantage of ‘holistic regulation.’

#### 5.3.3 Toxicity‐Modulating Synergy

Toxicity‐modulating synergy provides safety assurance for long‐term anti‐aging interventions. In SJZD, the core herb pair Atractylodes macrocephala–Poria cocos maintains intestinal barrier integrity and microbiota homeostasis. Atractylodes upregulates tight junction proteins (Occludin, ZO‐1) and enriches probiotics such as *Lactobacillus*. Poria polysaccharides repair the intestinal mucosa, reduce permeability, and suppress inflammatory responses [229]. Their combined use effectively alleviates gastrointestinal irritation that may be caused by tonic herbs such as licorice (Glycyrrhiza) [57]. Moreover, Poria cocos itself possesses anti‐aging potential: its polysaccharides enhance the activities of antioxidant enzymes (SOD and CAT) and regulate the insulin/insulin‐like growth factor 1 signalling pathway (daf‐2, age‐1, daf‐16), thereby delaying cellular and organismal aging [230]. This compatibility demonstrates the unity of toxicity reduction and efficacy enhancement, establishing a pharmacological foundation for the safe long‐term application of TCM formulas.

## 6 Methodological Innovations in TCM Anti‐Aging Research

### 6.1 Network Pharmacology and AI‐Driven Target Prediction

Network pharmacology and molecular docking technology have significantly promoted the development of TCM compound research by achieving systematic prediction of active ingredients, target identification, and pathway enrichment analysis. In recent years, its deep integration with artificial intelligence and machine learning has pushed TCM anti‐aging research from ‘ingredient screening’ to ‘target network prediction’. By integrating databases such as TCMSP with protein interaction networks, it is possible to systematically predict aging‐related signalling nodes regulated by multiple components in the compound. The screening and modelling of key targets (AMPK, TLR7/8) for the anti‐vascular aging effect of the representative BYHWD [158, 231]. Furthermore, a machine‐learning‐based high‐throughput screening model for senolytic compounds has been applied to large datasets, including the TCM bank, successfully identifying 614 potential senolytic compounds and 81 herbs with senolytic characteristics [232, 233]. The AI‐driven target prediction strategy can significantly narrow down the scope of experimental verification and provide rational hypotheses for the analysis of the mechanism of action of complex compounds.

Nevertheless, AI‐driven senolytic screening still has certain limitations. First, the training datasets for these models are predominantly derived from known compound libraries, which may introduce inherent biases toward structurally well‐characterized and well‐studied compounds, potentially underestimating the predictive capacity for structurally novel or non‐traditional pharmacologically active entities [234, 235, 236]. Second, current AI models face substantial challenges in predicting the systemic effects of complex herbal mixtures, as most algorithms are optimized for single‐compound activity prediction and are ill‐equipped to capture the synergistic effects arising from multi‐component interactions [237, 238]. To address these constraints, we propose that AI predictions should be tightly integrated with in vitro and in vivo experimental validation. Specifically, following primary AI screening, molecular docking against aging‐related protein structures [239, 240], such as SIRT1, mTOR, and NF‐KB, should be employed as a complementary interpretative tool, thereby enhancing the reliability of hit selection and providing structural rationales for subsequent experimental interrogation [241].

### 6.2 Multi‐Omics Integration and Aging Clock Assessment

Single omics is difficult to capture the overall effect of TCM compound multi‐target regulation of the aging network, and multi‐omics integration has become the core means to reveal its systemic mechanism. Transcriptomics, 16S rRNA sequencing, and untargeted metabolomics can reveal the enrichment regulation of specific pathways by the compound, its corrective effects on energy and amino acid metabolism disorders, and identify key protein targets. The most significant breakthrough is the construction of a proteomic aging clock based on ultra‐high‐sensitivity mass spectrometry, which can evaluate biological age across 12 tissue types and quantitatively assess the reversal effect of the compound on multi‐organ biological age [242]. For example, BZBC has been proven to reverse DNA methylation age in mice through this technology [18]. In addition, high‐resolution analytical chemistry techniques such as UPLC‐Q‐TOF‐MS and UPLC‐Q‐Orbitrap MS/MS have become indispensable tools for identifying and characterizing complex chemical components in TCM formulas. These high‐resolution mass spectrometry and multi‐omics platforms can comprehensively analyze compound ingredients and identify bioactive compounds, opening up a new path for standardized and quantitative evaluation of TCM anti‐aging efficacy.

### 6.3 Multi‐Level Model Biovalidation System

From molecular prediction to clinical translation, it is necessary to construct a validation ladder that spans different biological complexities. *C. elegans* is a major screening platform for lifespan determination, stress resistance testing, and genetic pathway validation, as demonstrated by studies on the autophagy mechanism of ZGP/YGP and activation of the JFG DAF‐16/HSF‐1/SKN‐1 pathway [117, 243]. The *Drosophila melanogaster* provides intermediate validation for complex behavioural and physiological assessments [244]. The D‐galactose‐induced aging model in rodents can comprehensively evaluate organ‐specific effects, while SAMP8 rapidly aging mice provide an accelerated aging phenotype for studying brain aging and cognitive function [113]. The natural aging model is most clinically relevant, but requires a longer research period. This progressive verification system ensures the reliability and clinical relevance of TCM anti‐aging findings.

## 7 Challenges and Future Perspectives

### 7.1 Chemical Complexity and Mechanism Analysis

The ‘multi‐component multi‐target’ characteristic of TCM prescriptions is not only an anti‐aging advantage, but also a core challenge in mechanism analysis and quality control. First, variations in geographical origin, harvest season, and processing methods can substantially alter the chemical composition and content of herbal materials, directly affecting pharmacological consistency and experimental reproducibility. For example, the levels of major bioactive compounds, such as ginsenosides and astragalosides, can vary severalfold depending on cultivation region, harvest time, and post‐harvest processing, including drying, steaming, or stir‐frying. These batch‐to‐batch discrepancies may underlie conflicting efficacy reports [245, 246]. To minimize such variability, rigorous quality control measures, including chromatographic fingerprinting and adherence to agricultural and manufacturing practices, are essential prerequisites. Second, different aging models, including D‐galactose‐induced, naturally aged, and SAMP8/SAMP6 rapid‐aging models, recapitulate distinct aspects of the aging process and may therefore exhibit differential sensitivity to a given formula [247, 248, 249]. Third, although core aging regulatory pathways, such as Insulin/IGF‐1 signalling, mitophagy, and mTOR‐mediated nutrient sensing, are evolutionarily conserved [250], species differences in drug‐metabolizing enzymes and target homology do exist [251, 252]. These differences do not invalidate cross‐species studies; rather, they underscore the necessity of multi‐species validation. Current TCM anti‐aging research generally follows a progressive validation hierarchy, moving from lower model organisms (e.g., *C. elegans* and *Drosophila*) to mammals (mice and rats) and ultimately to humans [253]. When a given formula, such as BZBC, demonstrates consistent anti‐aging effects across *C. elegans* , mice, and humans, the cumulative evidence gains substantially greater strength than that derived from any single model, suggesting that cross‐species validation has become a methodological cornerstone for translating TCM anti‐aging discoveries.

In the field of aging therapeutics, synthetic multi‐target agents have been emerging. Although these agents achieve potent intervention through high‐affinity binding, they are prone to off‐target effects on structurally related non‐target kinases or homologous proteins, and high‐affinity binding often triggers feedback‐driven compensatory activation, leading to cascading toxicities [254, 255]. For example, reported AMPK activators/mTOR inhibitors, despite suppressing thyroid cancer cell proliferation, also promote cell migration and lack data on multi‐organ and normal‐cell toxicity. PI3K/mTOR dual inhibitors have frequently been discontinued in clinical trials due to intolerable gastrointestinal and metabolic toxicities [256]. Chronic inhibition of mTORC1/mTORC2, while activating autophagy, impairs cell growth and tissue repair [257]. Consequently, the development of such agents is constrained by off‐target effects, long‐term toxicity, and lack of organ specificity. By contrast, TCM formulas operate under a fundamentally different paradigm: their active constituents exhibit relatively low plasma concentrations and modest target affinity, yet through multi‐component synergy they generate additive or cooperative effects, exerting ‘multi‐target fine‐tuning’ on both primary targets and bypass signalling to achieve network rebalancing without provoking feedback compensatory activation, thereby maintaining systemic homeostasis and multi‐organ function [9, 258]. Moreover, many herbal medicines and formulas display organ preference and differential regulatory properties, potentially reducing off‐target risks at the organ and cell‐type levels [259, 260, 261].

The aforementioned characteristics render conventional approaches inadequate for comprehensively capturing formula‐mediated regulation of aging networks, resulting in an unclear material basis and ambiguous target engagement, which directly constrains the reliability of clinical translation. To this end, in the future, artificial intelligence, network pharmacology, and multi‐omics technologies should be systematically integrated to construct a multidimensional regulatory map of ‘components targets aging phenotype’, and priority should be given to applying proteomics aging clock quantification formulas to reverse the biological age of organs. This strategy not only elucidates the mechanism, but also provides a basis for establishing biomarkers associated with clinical endpoints, promoting the transformation of TCM prescriptions from empirical summary to mechanism support.

### 7.2 Clinical Evidence Gap

The current clinical research on TCM anti‐aging generally has problems such as small sample size and subjective outcome indicators, and rarely uses internationally recognized aging biomarkers (such as epigenetic clock, circulating aging‐related protein GAS6, GPNMB) as efficacy indicators, leading to a lack of high‐quality RCT evidence and becoming a bottleneck for the entry of prescriptions into mainstream anti‐aging clinical practice. The future breakthrough path lies in conducting precise clinical trials guided by biomarkers: on the one hand, using blood proteomic characteristics or TCM constitution typing to screen out trial populations that may be more sensitive to intervention; on the other hand, using quantitative indicators such as epigenetic age reversal and SA‐β‐gal positivity rate reduction as core secondary endpoints can shorten the trial period, improve the level of evidence, and promote the clinical translation and regulatory registration of TCM prescriptions.

### 7.3 Clinical New Drug Conversion Disorder

A large number of promising TCM anti‐aging formulas are stuck in basic research, making it difficult to achieve clinical translation into new drugs. Key obstacles include poor chemical batch consistency, lack of standardized drug efficacy substance lists, blank long‐term safety and drug interaction data, and unclear positioning of product attributes (drugs/functional foods). To overcome these obstacles, it is necessary to establish a transformation path of ‘chemical quality control mechanism verification clinical evaluation’: at the chemical level, establish high‐resolution mass spectrometry to construct prescription fingerprint spectra to ensure quality control; at the mechanism level, a multi‐level model is used to verify the locking effect mechanism and the active component group; at the clinical translation level, priority is given to accumulating clinical data based on functional foods for safer medicinal and food homologous formulas, while for formulas with definite therapeutic effects, aging biomarkers are used as alternative endpoints to promote randomized controlled trials. In summary, this comprehensive framework may provide a practical approach for advancing TCM‐based anti‐aging interventions from laboratory research to clinical application and product development.

## 8 Conclusion

TCM formulas combat aging through a paradigm fundamentally distinct from synthetic drugs: multi‐component, multi‐target, and multi‐pathway synergy that mirrors the systemic complexity of aging itself. These formulas are rooted in classical theories represented by ‘Kidney Essence Depletion Theory’, ‘Kidney Deficiency and Blood Stasis Theory’, *‘Qi‐Blood Theory’, ‘Spleen‐Stomach Theory’*, and have been validated by the 14 modern hallmarks of aging; these formulas restore systemic balance rather than merely intervening in isolated pathways. Recent breakthroughs in proteomic aging clocks, circulating senescence biomarkers, and multi‐omics integration have enabled quantitative assessment of biological age reversal, providing a robust technical framework for evaluating TCM formula efficacy. Methodologically, AI‐driven compound screening, high‐resolution mass spectrometry, and multi‐omics integration have transformed empirical formulas into testable mechanistic hypotheses. Nevertheless, challenges remain: chemical complexity, lack of standardized quality markers, incomplete mechanistic elucidation, and a critical gap in large‐scale randomized controlled trials. The path forward demands a precision paradigm that integrates proteomic signatures with TCM constitution typing for patient stratification, employs epigenetic age reversal as a core endpoint, and pushes traditional formulas toward regulatory‐grade clinical trials. In summary, bridging TCM gerontology with modern aging biology will transform empirical wisdom into clinically validated strategies—not merely to extend lifespan, but to compress morbidity and extend healthspan for the global aging population.

## Author Contributions

**Chenrong Jin:** conceptualization, writing – original draft. **Ding Du:** conceptualization, writing – review and editing. **Xiaorui Yu:** supervision, writing – review and editing. **Xiaoyan Ji:** supervision, writing – review and editing. **Xiuyun Zhang:** writing – original draft. **Anning Li:** writing – original draft. **Shuang Zu:** investigation, supervision. **Bo Qu:** investigation, supervision. **Daqing Zhao:** project administration. **Dianpeng Wu:** project administration, writing – review and editing. **Meichen Liu:** conceptualization, project administration, writing – review and editing.

## Funding

This work was supported by Jilin Province Changbai Talent Program youth top‐notch talent project (grant number 20240718181); Changchun University of Traditional Chinese Medicine Affiliated Hospital Metabolic Disease Research Special Project (grant numbers DXZX‐06‐01, DXZX‐06‐02).

## Conflicts of Interest

The authors declare no conflicts of interest.

## Data Availability

Data sharing not applicable to this article as no datasets were generated or analysed during the current study.
